# Behavioral compliance with preventive health measures for students with and without hearing disability during COVID-19 pandemic: A cross-sectional study

**DOI:** 10.3389/fpubh.2022.911671

**Published:** 2022-09-16

**Authors:** Ying Yang, Yulu Liu, Yanan Xiao, Chengyi Qu, Philip H.-S. Jen

**Affiliations:** ^1^Department of Hearing and Speech Rehabilitation, Binzhou Medical University, Yantai, China; ^2^Department of Epidemiology, Shanxi Medical University, Taiyuan, China; ^3^Division of Biological Sciences and Interdisciplinary Neuroscience Program, University of Missouri-Columbia, Columbia, MO, United States

**Keywords:** preventive health measures, COVID-19, health behaviors, risk factors, hearing disability

## Abstract

**Background:**

Hearing loss affects over 1.5 billion individuals worldwide. Their disability and limited access to the coronavirus (COVID-19) pandemic information make them suffer a greater degree than ordinary people. However, the quantitative studies on the implementation of behavior compliance with preventive health measures for vulnerable groups such as people with hearing disability were limited. The purpose of this study was to explore the compliance with pandemic-related protective health measures among people with hearing disability.

**Design:**

A cross-sectional survey, population-based cohort study of students aged 12–26 years with and without hearing disability was conducted. Behavioral compliance with preventive health measures was collected from the general education institutions and special education schools using an online questionnaire. Logistic regression and structural equation model were used to determine the associations among the demographic variables, different degrees of mental health status and psychological impacts, and preventive health behaviors.

**Results:**

Among 1,589 participants, 485 (30.5%) students are with hearing disability (SHD), and 1,104 (69.5%) students with normal hearing (SNH). The SHD has a significantly lower degree of behavioral compliance with the preventive health measures than SNH has. Hearing disability and anxiety [odds ratio (OR) = 1.54–1.76, *p* < 0.05] are risk factors for avoiding sharing of utensils during mealtime. Hearing disability, male sex, father's education level, mother's profession, bedtime after 11:00 p.m., anxiety, and depression (OR = 1.45–2.95, *p* < 0.05) are risk factors for hand hygiene. Male sex (OR = 2.13, *p* < 0.001) is risk factor and being aged below 18 years old (OR = 0.62, *p* = 0.03) is protective factor for wearing masks. Exercise (OR = 0.32–0.70, *p* < 0.01) is the most protective factor for preventive health behaviors. Mediating effect of mental health status and psychological impacts between hearing level and the compliance with the preventive health measures was −0.044 (95% CI: −0.068 to −0.027).

**Conclusions:**

To reduce the risk of contraction, update pandemic information, essential communication services, extra assistance, and support should be provided to these disabled persons who are more susceptible to a public health emergency.

## Introduction

Since the identification of several novel coronavirus (COVID-19) infection cases in December 2019 in Wuhan, Hubei Province, the worldwide spread of the pandemic has created a global health crisis (1). According to the World Health Organization (WHO), as of 12 March 2022, there have been 460,280,168 infected patients and 6,050,018 deaths caused by the COVID-19 pandemic (2).

This fatal COVID-19 is caused by the SARS-CoV-2 virus which transmits through respiratory droplets and close contact with people of all ages (3). The virus has an incubation period between 2 days and 2 weeks and its transmission can be asymptomatic (4).

Since the outbreak of the pandemic, the Chinese government has immediately implemented strict preventive health measures across the country (5). The WHO has also announced several preventive health measures including washing hands with soap and water, using an alcohol-based hand rub, covering the nose and mouth during coughing or sneezing, using utensils separately, and wearing a mask during social contact (6).

It has been shown that adequate preventive health measures aimed at vulnerable populations can effectively block the spread of respiratory diseases (7). In addition to preventing the pandemic spread, medical-grade masks can effectively filter out pathogens (8). Further study has also shown that mass masking and general hygiene at the early stage of the COVID-19 pandemic produces a 50% decline in infectious respiratory diseases (9). The spread of the pandemic, strict lockdown measures, and heavy economic burdens have produced the risk of death and enormous psychological impacts on the mental health of the population (10). However, the challenges faced by vulnerable groups are even more acute. Specifically, people with disabilities have the same healthcare needs as those without disabilities such as access to vaccinations or personal protection (11, 12). In addition, people with disabilities may require access to specific specialist services such as rehabilitation and assistive devices (12). Therefore, the need for healthcare services may be higher among people with disabilities, but their access to these services is poorer than for people without disabilities (13). Compared to the general population, individuals with disabilities have less access to healthcare services and more barriers to communication and are thus more likely to have a higher risk of depression, lower life satisfaction, and enhanced loneliness (14, 15).

The COVID-19 pandemic and its associated restrictions have posed challenges, especially to individuals with disabilities. Indeed they face barriers to implementing basic health measures and may have a higher risk of contracting COVID-19 (16). Individuals with disabilities make up about 15% of the global population, and it is important to include information on disabilities in the assessment of pandemic effects (17). The WHO has stated that additional considerations from governments, healthcare systems, disability service providers, institutional settings, and the disability community are needed for people with disabilities during the COVID-19 (18).

In particular, individuals with a hearing disability have poorer mental health than the general population and are highly vulnerable to major emergencies in terms of preparing for, responding to, and recovering from emergencies (19, 20). People with hearing disability often use gestures, while relying on visual information to enhance the comprehension of spoken messages to achieve communication (21). Measures against COVID-19 have significantly changed communication strategies, most people with hearing disability had difficulty in auditory communication with people wearing masks, especially in noisy surroundings or with physical distancing (22). Furthermore, a study has shown that hearing devices (i.e., hearing aids and cochlear implants) and speech services for students with hearing disability (SHD) that are essential for recovering or restoring patients' communication skills have not been consistently accessible during COVID-19 (23). The psychological distress of these vulnerable persons has been further accentuated because of limitations in communication, delayed access, and comprehension of the updated pandemic situation (23). Indeed, our previous study has shown that SHD suffers from a higher degree of mental stress and psychological distress than peer health groups during the COVID-19 pandemic (24).

On the other hand, a recent study has shown that the psychological status of persons is significantly associated with the preventive health measures taken during the pandemic (25). This is substantiated by the report that measures taken to prevent COVID-19 transmission have a protective psychological effect in the early stages of the pandemic outbreak (26). Furthermore, individual and social variables can influence compliance behavior with the preventive health measures during the COVID-19 pandemic (27).

All these studies prompt us to hypothesize that behavioral compliance with the preventive health measures during the COVID-19 pandemic would be different between ordinary and disabled persons. To test our hypothesis, we conducted a cross-sectional study of the behavioral compliance with the preventive measures between SHD and students with normal hearing (SNH) in multiple Chinese cities affected by the COVID-19 pandemic. Specifically, we statistically compared their degree of compliance with the preventive health measures to multiple demographic variables, mental health status and psychological impacts induced during the COVID-19 pandemic. Furthermore, we explore the relationship between hearing level, mental health status and psychological impacts, and preventive health behaviors.

## Materials and methods

### Study design and study population

The survey was conducted from 15 June 2020 to 23 November during the COVID-19 pandemic recurrence in 21 provinces (Liaoning, Henan, Heilongjiang, etc.) and 1 municipality (Beijing) in China. All data were collected using the cross-sectional survey design and snowball sampling method through Questionnaire Star (https://www.wjx.cn/) survey platform. Specifically, these participants were from middle schools, high schools, and universities (Binzhou Medical University; Liaoning Special Education Teachers College; Harbin Institute of Special Education, China, etc.) and special education schools (Yantai, Shenyang, Harbin, etc.). The study was approved by the Institutional Review Board of Binzhou Medical University (BMU-IRB-2020-54).

### Study tool

The information contained in the questionnaire includes the following categories.

(1) Demographic data including gender, age, educational background, parents' profession and education level, family living status, communication strategies, satisfaction with current communication mode, bedtime, physical activity, and information on hearing loss based on their previous diagnosis in otolaryngology;
(2) Data on compliance with four preventive health measures including avoiding sharing of utensils (e.g., chopsticks) during mealtime, washing hands with soap and water, washing hands immediately after coughing or sneezing, rubbing the nose, and wearing a mask with or without the pandemic symptoms. Participants rated on each item using a 5-point Likert scale (0 = “never”; 4 = “always”);
(3) The mental health status and psychological impacts of the participants were assessed using the Chinese version of the Depression Anxiety and Stress 21 (DASS-21) scale and the Impact of Events Scale-Revised (IES-R) (24). The suggested cut-off scores for detecting symptoms of major stress, anxiety, depression, and IES-R are 14, 7, 9, and 23, respectively.

### Statistical analysis

The prevention scores for these four basic health measures derived from SHD and SNH were compared using the following tests for statistical significance. We defined a prevention score of >2 as a low-risk score and ≤ 2 as a high-risk score. We first calculated the proportion of high-risk and low-risk students in each group. We then used univariate analysis to statistically compare the distribution of students with high-risk scores to students with low-risk scores between SHD and SNH in demographic variables, different degrees of mental health status and psychological impacts. All the variables were subsequently analyzed by a multivariable regression model using SPSS Statistic 21.0 (IBM Corporation, New York, NY, United States) at a significance level of *p* < 0.001–0.05.

The structural equation model was conducted using the software Amos 24.0 (IBM Corp., Chicago, IL, USA). According to the hearing thresholds for the better ear, all respondents were divided into five hearing levels: normal hearing (≤ 25 dB HL), mild hearing loss (26–40 dB HL), moderate hearing loss (41–60 dB HL), severe hearing loss (61–80 dB HL), and profound hearing loss (≥81 dB HL). (I) Pearson correlation analysis was used to explore the relationship among the hearing level, mental health status and psychological impacts as well as the prevention scores of four preventive health measures. Then, we performed the linear regression model to examine the relationship between the hearing level and the scores of four preventive measures to explore whether there was a dose-dependent effect between hearing level and compliance with preventive measures. (II) The Amos 24.0 software was used to establish the structural equation model of the relationship among three variables: hearing level, mental health status and psychological impacts, and compliance with four preventive health measures. (III) The bootstrap method was used to determine the mediating effect of mental health status and psychological impacts between the level of hearing and compliance with four preventive health measures.

## Results

Of the 1,697 students with and without hearing loss collected from 25 provinces and 2 municipalities during the study period, 1,589 were available for analysis after excluding unreliable information (i.e., those participants outside the age range between 12 and 26 years old, incomplete or unclear information, etc.) with the questionnaire effective rate reaching 96.2% (97.4% for SNH, 93.4% for SHD). The students who reported any neurological disorders or cognitive impairment were excluded. There were 1,005 (63%) female participants and 584 (37%) male participants with age ranging from 12 to 26 years [12–14 years (421 [27%]), 15–18 years (628 [40%]), 19–22 years (519 [33%]), and 22–26 years (17 [1%])].

There are 1,104 (69.5%) SNH and 485 (30.5%) SHD. There are 373 (76.9%) SHD with profound hearing loss (≥ 81 dB HL in the better ear) and 361 (74.4%) SHD fitted with hearing aids or cochlear implantation. The communication strategies for 380 (78.4%) SHD is the gesture, lip-language, gesture plus lip-language, and word.

### Comparison of demographic variables, mental health status and psychological impacts with the compliance with four health measures between SNH and SHD

**Figure 1:**
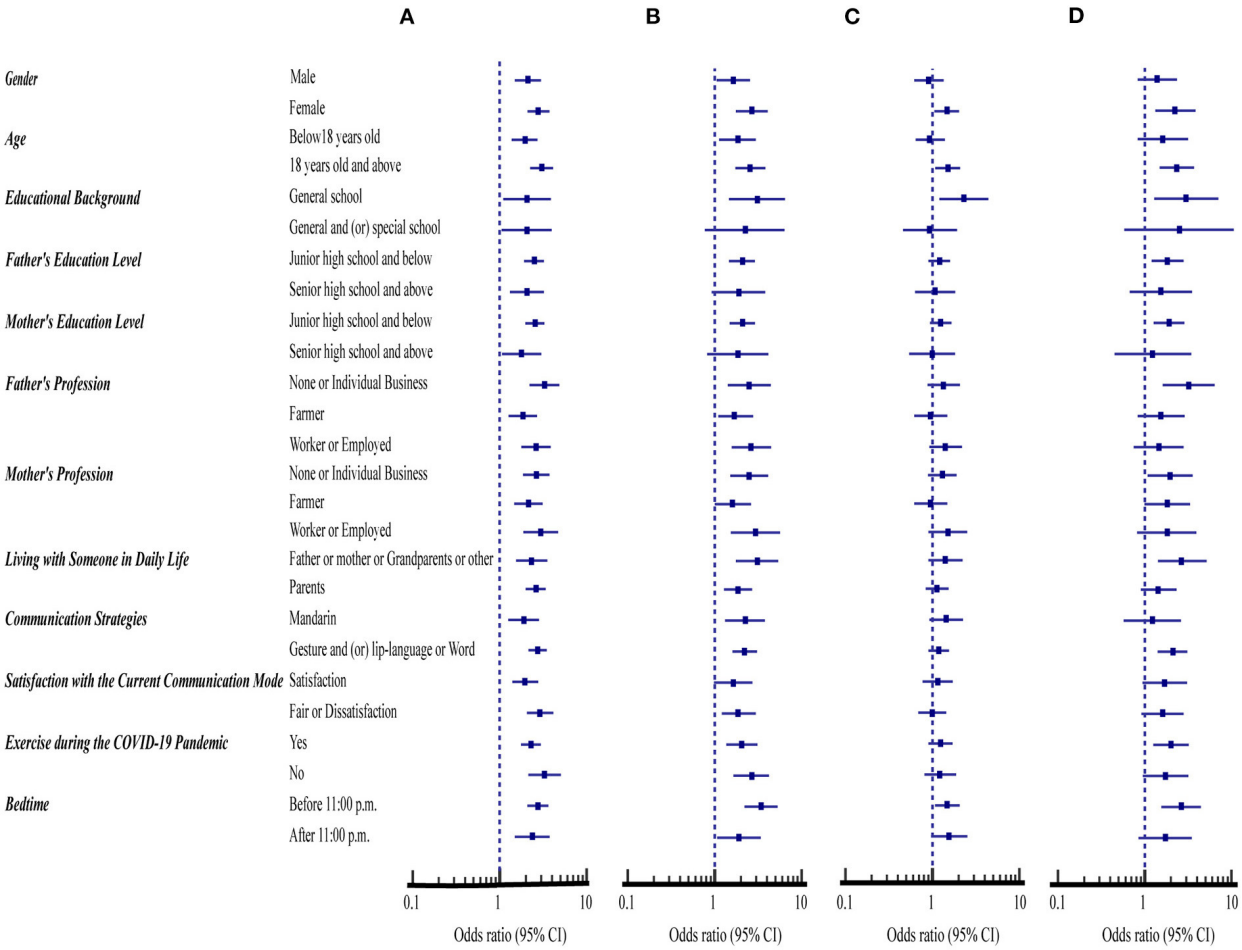
Comparison of demographic variables with the compliance with four health measures between students with normal hearing (SNH, *n* = 1,104) and with hearing disability (SHD, *n* = 485). **(A)** Avoiding sharing of utensils during mealtime. **(B)** Washing hands with soap and water. **(C)** Washing hands immediately after coughing, or sneezing, rubbing the nose. **(D)** Wearing masks with or without the pandemic symptoms.

Figure 1 compares the association of the demographic variables with the high-risk scores (≤ 2) for four preventive health measures between SNH and SHD. The percent of participants with a high risk of ≤ 2 prevention score was significantly higher for the SHD than for the SNH in all demographic variables [odds ratio (OR) = 1.78–3.28, 95% CI: 1.05 to 5.04, *p* < 0.05] (Figure 1A, Table 1 first column).

**Table 1:** The percentage of demographic variables with health behavior of ≤ 2 high-risk prevention scores among students with and without hearing disability.

	**Group**	**Avoiding sharing of utensils during mealtime**	**Washing hands with soap and water**	**Washing hands immediately after coughing, or sneezing, rubbing the nose**	**Wearing masks with or without the pandemic** **symptoms**
**Gender**					
Male	SNH	30.4%	13.7%	26.8%	9.9%
		(111)	(50)	(98)	(36)
	SHD	47.9%	20.5%	25.1%	13.2%
		(105)	(45)	(55)	(29)
Female	SNH	26.4%	7.4%	19.4%	4.6%
		(195)	(55)	(143)	(34)
	SHD	50.0%	17.7%	25.9%	9.8%
		(133)	(47)	(69)	(26)
**Age**					
Below 18 years old	SNH	30.8%	10.4%	23.8%	5.2%
		(101)	(34)	(78)	(17)
	SHD	46.2%	17.4%	22.7%	8.1%
		(114)	(43)	(56)	(20)
18 years old and above	SNH	26.4%	9.1%	21.0%	6.8%
		(205)	(71)	(163)	(53)
	SHD	52.1%	20.6%	28.6%	14.7%
		(124)	(49)	(68)	(35)
**Educational background**					
General school	SNH	27.5%	9.5%	21.7%	6.4%
		(292)	(101)	(230)	(68)
	SHD	43.9%	24.4%	39.0%	17.1%
		(18)	(10)	(16)	(7)
General and (or) special school	SNH	32.6%	9.3%	25.6%	4.7%
		(14)	(4)	(11)	(2)
	SHD	49.5%	18.5%	24.3%	10.8%
		(220)	(82)	(108)	(48)
**Father's education level**					
Junior high school and below	SNH	30.3%	11.5%	23.7%	7.2%
		(203)	(77)	(159)	(48)
	SHD	51.8%	21.1%	27.1%	12.4%
		(197)	(80)	(103)	(47)
Senior high school and above	SNH	23.8%	6.5%	18.9%	5.1%
		(103)	(28)	(82)	(22)
	SHD	39.0%	12.4%	20.0%	7.6%
		(41)	(12)	(21)	(8)
**Mother's education level**					
Junior high school and below	SNH	29.6%	10.9%	22.4%	6.9%
		(219)	(81)	(166)	(51)
	SHD	51.6%	20.4%	26.5%	12.3%
		(210)	(83)	(108)	(50)
Senior high school and above	SNH	23.9%	6.6%	20.6%	5.2%
		(87)	(24)	(75)	(19)
	SHD	35.9%	11.5%	20.5%	6.4%
		(28)	(9)	(16)	(5)
**Father's profession**					
None or Individual business	SNH	24.1%	7.6%	21.5%	4.4%
		(82)	(26)	(73)	(15)
	SHD	50.9%	17.2%	27.0%	12.9%
		(83)	(28)	(44)	(21)
Farmer	SNH	33.2%	12.7%	23.7%	7.6%
		(105)	(44)	(75)	(24)
	SHD	47.8%	19.7%	23.0%	11.2%
		(85)	(35)	(41)	(20)
Worker or Employed	SNH	26.6%	8.7%	20.8%	6.9%
		(119)	(39)	(93)	(31)
	SHD	48.6%	20.1%	27.1%	9.7%
		(70)	(29)	(39)	(14)
**Mother's profession**					
None or Individual business	SNH	24.5%	7.9%	22.1%	5.8%
		(102)	(33)	(92)	(24)
	SHD	46.0%	17.7%	27.0%	10.7%
		(99)	(38)	(58)	(23)
Farmer	SNH	33.1%	14.2%	23.5%	7.5%
		(110)	(47)	(78)	(25)
	SHD	51.5%	21.1%	22.8%	12.9%
		(88)	(36)	(39)	(22)
Worker or Employed	SNH	26.4%	7.0%	19.9%	5.9%
		(94)	(25)	(71)	(21)
	SHD	51.5%	18.2%	27.3%	10.1%
		(51)	(18)	(27)	(10)
**Living with someone in daily life**					
Father or mother or Grandparents or other	SNH	28.5%	9.3%	22.4%	6.9%
		(70)	(23)	(55)	(17)
	SHD	48.1%	24.1%	29.0%	16.7%
		(78)	(39)	(47)	(27)
Parents	SNH	27.5%	9.6%	21.7%	6.2%
		(236)	(82)	(186)	(53)
	SHD	49.5%	16.4%	23.8%	8.7%
		(160)	(53)	(77)	(28)
**Communication strategies**					
Mandarin	SNH	27.7%	9.5%	21.8%	6.3%
		(306)	(105)	(241)	(70)
	SHD	41.9%	19.0%	28.6%	7.6%
		(44)	(20)	(30)	(8)
Gesture and (or)	SHD	51.1%	18.9%	24.7%	12.4%
lip-language or word		(194)	(72)	(94)	(47)
**Satisfaction with the current communication mode**					
Satisfaction	SNH	27.8%	8.4%	20.1%	5.8%
		(235)	(71)	(170)	(49)
	SHD	43.2%	13.0%	22.5%	9.5%
		(73)	(22)	(38)	(16)
Fair or Dissatisfaction	SNH	27.3%	13.1%	27.3%	8.1%
		(71)	(34)	(71)	(21)
	SHD	52.2%	22.2%	27.2%	12.3%
		(165)	(70)	(86)	(39)
**Exercise during the COVID-19 pandemic**					
Yes	SNH	26.4%	6.8%	16.8%	5.0%
		(215)	(55)	(137)	(41)
	SHD	45.1%	13.0%	20.0%	9.6%
		(160)	(46)	(71)	(34)
No	SNH	31.4%	17.2%	35.9%	10.0%
		(91)	(50)	(104)	(29)
	SHD	60.0%	35.4%	40.8%	16.2%
		(78)	(46)	(53)	(21)
**Bedtime**					
Before 11:00 p.m.	SNH	25.9%	6.2%	17.2%	4.5%
		(133)	(32)	(88)	(23)
	SHD	49.0%	18.5%	23.5%	11.0%
		(196)	(74)	(94)	(44)
After 11:00 p.m.	SNH	29.3%	12.4%	25.9%	8.0%
		(173)	(73)	(153)	(47)
	SHD	49.4%	21.2%	35.3%	12.9%
		(42)	(18)	(30)	(11)

SNH, students with normal hearing; SHD, students with hearing disability.

Excluding the general and or special school educational background, parents' education level with senior high school and above, and satisfaction with the current communicative mode, the percent of participants with a high risk of ≤ 2 prevention score in the preventive measure of washing hands with soap and water was significantly higher for the SHD than for the SNH in all other demographic variables (OR = 1.62–3.41, 95% CI: 1.00 to 6.44, *p* < 0.05) (Figure 1B, Table 1 second column).

Furthermore, the percent of participants with a high risk of ≤ 2 prevention score in the preventive measure of washing hands immediately after coughing, or sneezing, rubbing the nose was significantly higher for SHD in four demographic variables, including females, aged 18 years old and above, general school educational background, bedtime before 11:00 p.m. (OR = 1.46–2.31, 95% CI: 1.05 to 4.40, *p* < 0.05) (Figure 1C, Table 1 third column). Also, < 41.0% of SNH and SHD for both hand hygiene had ≤ 2 high-risk prevention scores in all demographic variables when compared with < 60.0% of SNH and SHD for avoiding sharing of utensils during mealtime (Table 1 first, second, third columns).

Respectively, the percent of SNH and SHD that had ≤ 2 high-risk prevention scores of wearing masks were < 10% and 17% in all demographic variables studied. However, the percent of this preventive measure was significantly higher for the SHD than for the SNH in nine demographic variables, including females, aged 18 years old and above, general school educational background, parents' education level with junior high school and below, parents' profession with individual and non-working, living with father or mother, grandparents or other, communicative means with gesture and (or) lip-language or word, exercising during the COVID-19 pandemic, bedtime before 11:00 p.m. (OR = 1.83–3.20, 95% CI: 1.08 to 7.03, *p* < 0.05) (Figure 1D, Table 1 fourth column).

**Figure 2:**
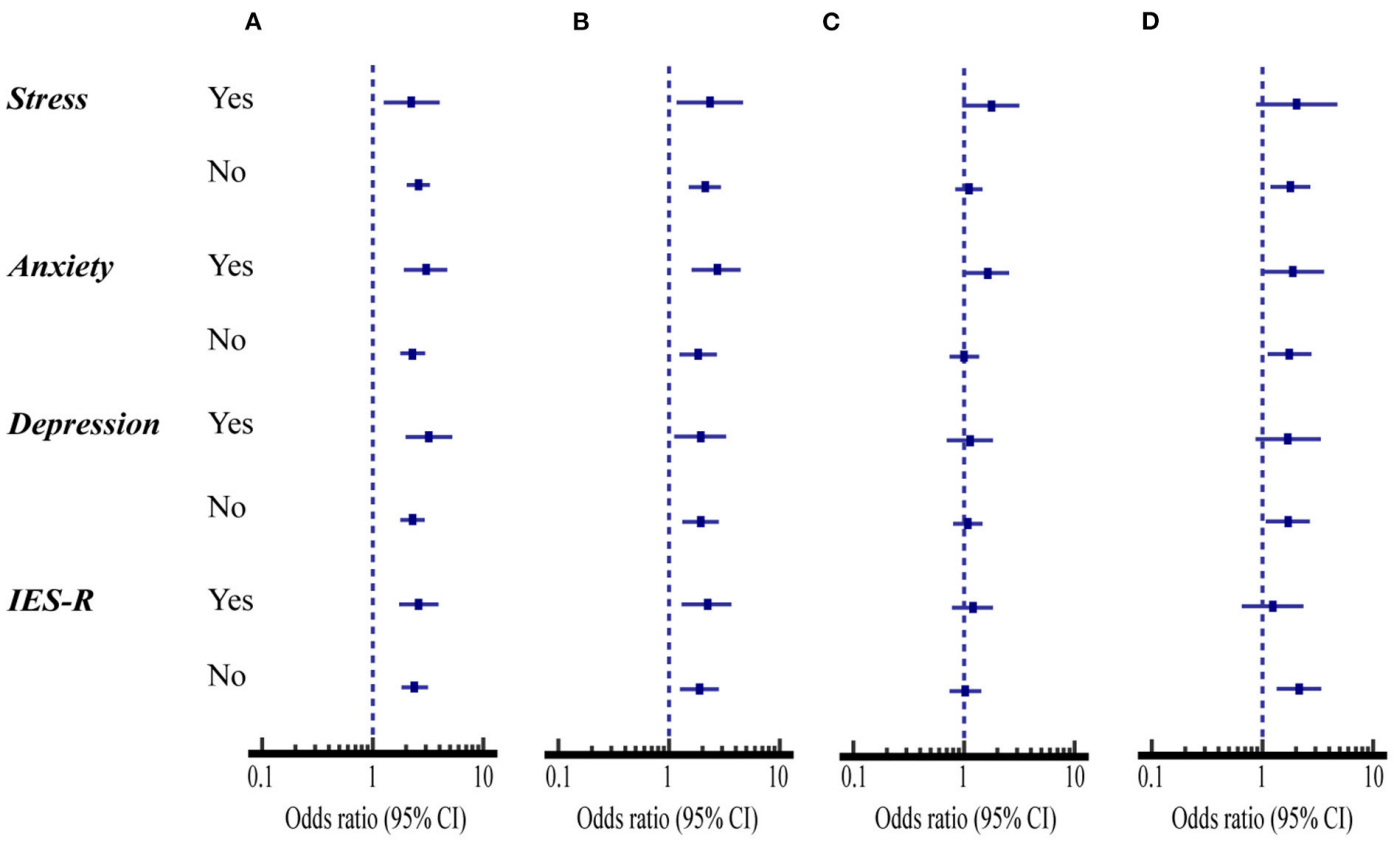
Comparison of mental health status and psychological impacts with the compliance with four health measures between students with normal hearing (SNH, *n* = 1,104) and with hearing disability (SHD, *n* = 485). **(A)** Avoiding sharing of utensils during mealtime. **(B)** Washing hands with soap and water. **(C)** Washing hands immediately after coughing, or sneezing, rubbing the nose. **(D)** Wearing masks with or without the pandemic symptoms.

Figure 2 compares the association of mental health status and psychological impacts with the preventive health measures between SNH and SHD. The percent of the SHD that had ≤ 2 high-risk prevention scores were significantly higher for avoiding sharing utensils during mealtime and washing hands with soap and water than the SNH regardless of whether their mental health status and psychological impacts on stress, anxiety, depression, and IES-R or not (OR = 1.83–3.21, 95% CI: 1.12 to 5.22, *p* < 0.05) (Figures 2A,B, Table 2 first and second columns).

**Table 2:** The percentage of mental health status and psychological impacts with health behavior of ≤ 2 high-risk prevention scores among students.

	**Group**	**Avoiding sharing of utensils during mealtime**	**Washing hands with soap and water**	**Washing hands immediately after coughing, or sneezing, rubbing the nose**	**Wearing masks with or without the pandemic** **symptoms**
**Stress** ^**a**^					
Yes	SNH	26.6%	14.1%	25.0%	8.6%
		(34)	(18)	(32)	(11)
	SHD	44.8%	27.6%	36.8%	16.1%
		(39)	(24)	(32)	(14)
No	SNH	27.9%	8.9%	21.4%	6.0%
		(272)	(87)	(209)	(59)
	SHD	50.0%	17.1%	23.1%	10.3%
		(199)	(68)	(92)	(41)
**Anxiety** ^**b**^					
Yes	SNH	31.4%	15.7%	29.6%	9.4%
		(70)	(35)	(66)	(21)
	SHD	57.9%	33.1%	40.6%	16.5%
		(77)	(44)	(54)	(22)
No	SNH	26.8%	7.9%	19.9%	5.6%
		(236)	(70)	(175)	(49)
	SHD	45.7%	13.6%	19.9%	9.4%
		(161)	(48)	(70)	(33)
**Depression** ^**c**^					
Yes	SNH	27.7%	19.5%	34.6%	10.7%
		(44)	(31)	(53)	(17)
	SHD	55.1%	31.6%	37.5%	16.9%
		(75)	(43)	(51)	(23)
No	SNH	27.7%	7.8%	19.7%	5.6%
		(262)	(74)	(186)	(53)
	SHD	46.7%	14.0%	20.9%	9.2%
		(163)	(49)	(73)	(32)
**IES-R** ^**d**^					
Yes	SNH	28.6%	13.3%	29.5%	9.5%
		(60)	(28)	(62)	(20)
	SHD	51.0%	25.0%	33.3%	11.5%
		(98)	(48)	(64)	(22)
No	SNH	27.5%	8.6%	20.0%	5.6%
		(246)	(77)	(179)	(50)
	SHD	47.8%	15.0%	20.5%	11.3%
		(140)	(44)	(60)	(33)

IES-R, Impact of Events Scale-Revised.

^a^Yes, 15–42 scores; No, less than or equal to 14 scores.

^b^Yes, 8–42 scores; No, less than or equal to 7 scores.

^c^Yes, 10–42 scores; No, less than or equal to 9 scores.

^d^Yes, 24–88 scores; No, less than or equal to 23 scores.

However, the percent of washing hands immediately after coughing, sneezing, rubbing the nose was only significantly higher for the SHD than for the SNH when they suffered anxiety (OR = 1.63, 95% CI: 1.04 to 2.55, *p* < 0.05) (Figure 2C, Table 2 third column).

When the students suffered anxiety, the percent of the SHD that had ≤ 2 high-risk prevention scores for wearing masks with or without the pandemic symptoms was significantly higher for the SHD than for the SNH (OR = 1.91, 95% CI: 1.00 to 3.62, *p* < 0.05). On the other hand, when the students did not suffer stress, anxiety, depression, and IES-R, the percent of the compliance with this health measure was significantly lower for the SHD than for the SNH (OR = 1.70–2.14, 95% CI: 1.08 to 3.40, *p* < 0.05) (Figure 2D, Table 2 fourth column).

### Association of demographic variables, mental health, and psychological status with compliance with four health measures in SNH and SHD

**Figure 3:**
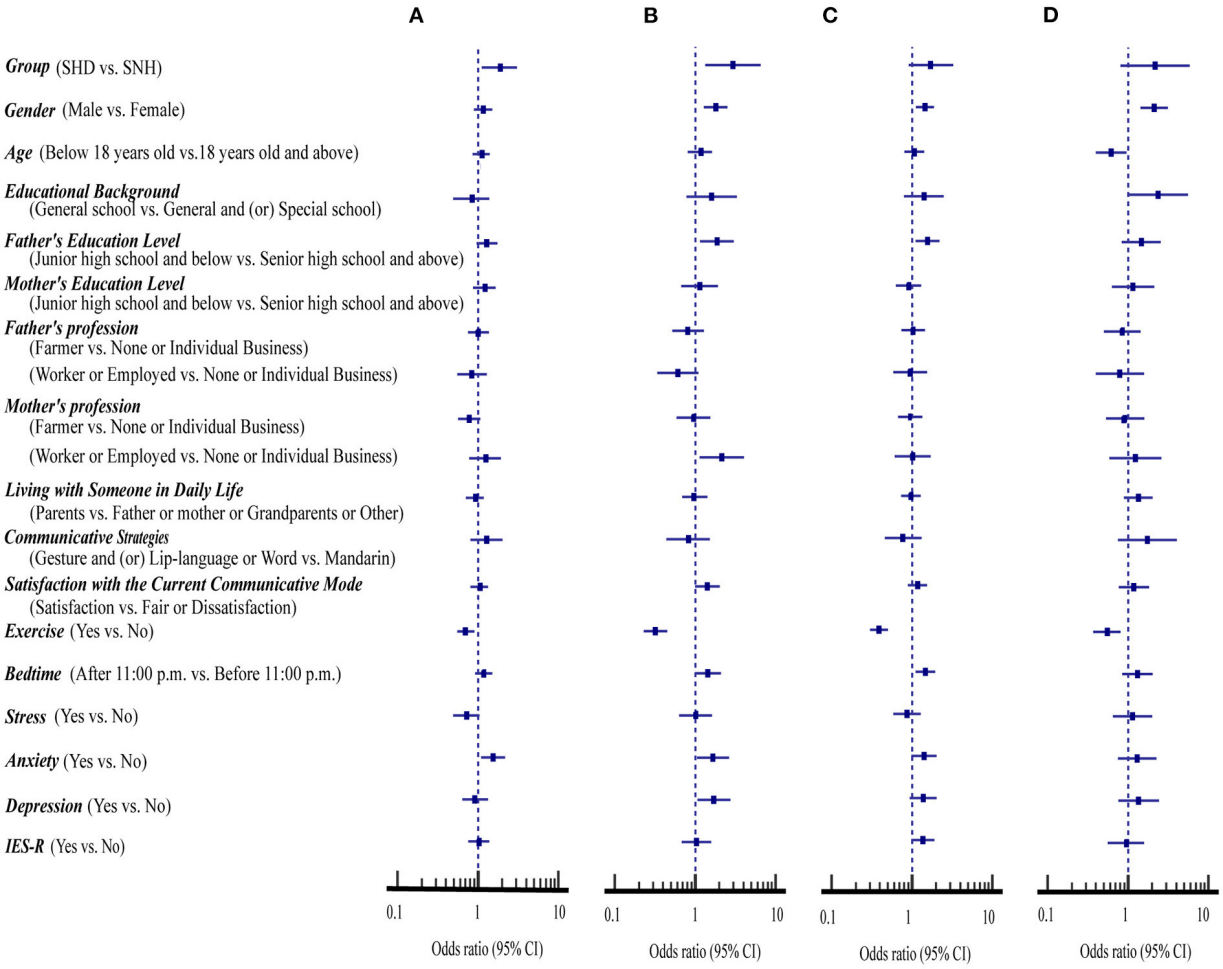
Association of demographic variables, mental health status and psychological impacts with the compliance with four health measures in students with and without hearing disability (*n* = 1,589). **(A)** Avoiding sharing of utensils during mealtime. **(B)** Washing hands with soap and water. **(C)** Washing hands immediately after coughing, or sneezing, rubbing the nose. **(D)** Wearing masks with or without the pandemic symptoms.

Figure 3 summarizes the association of demographic variables, mental health status and psychological impacts with the prevention scores for all students. Multivariate analysis showed that hearing disability and anxiety status (OR = 1.54–1.76, 95%CI: 1.00 to 3.07, *p* < 0.05) were related to an increased risk of sharing of utensils during mealtime while exercise was a protective factor (OR = 0.70, 95%CI: 0.55 to 0.90, *p* < 0.01) for avoiding sharing of utensils during mealtime (Figure 3A).

In addition, hearing loss, male sex, father's education level of junior high school and below, mother's profession of worker or employed, anxiety, and depression were related to increased risks of the compliance with washing hands with soap and water (OR = 1.67–2.95, 95%CI: 1.06 to 6.52, *p* < 0.05) (Figure 3B).

Furthermore, male sex, father's education level of junior high school and below, and bedtime after 11:00 p.m. were risk factors for the compliance with washing hands immediately after coughing, or sneezing, rubbing the nose (OR = 1.45–1.56, 95%CI: 1.11 to 2.20, *p* < 0.05) (Figure 3C). The exercise was the only protective factor for hand hygiene (OR = 0.32–0.39, 95%CI: 0.23 to 0.50, *p* < 0.001).

Similarly, male sex (OR = 2.13, 95%CI: 1.44 to 3.14, *p* < 0.001) was a risk factor while exercise and being age below 18 years old (OR = 0.55–0.62, 95%CI: 0.37 to 0.96, *p* < 0.05) were the protective factors for wearing masks with or without the pandemic symptoms (Figure 3D).

### Correlation analysis of hearing level with mental health, psychological impacts, and compliance with the preventive health measures

Table 3 showed that the level of the hearing was positively correlated with mental health and psychological impacts (*r* = 0.068 to 0.277, *p* < 0.01). Meanwhile, The level of the hearing was negatively correlated with preventive scores of avoiding sharing of utensils during mealtime, washing hands with soap and water, and wearing masks with or without the pandemic symptoms (*r* = −0.202 to −0.059, *p* < 0.05).

**Table 3:** Correlation analysis of the hearing level, mental health status and psychological impacts, and the prevention scores of four preventive health measures.

**Variable**	**Stress**	**Anxiety**	**Depression**	**IES-R**	**Avoiding sharing of utensils during mealtime**	**Washing hands with soap and water**	**Washing hands immediately after coughing, or sneezing, rubbing the nose**	**Wearing masks with or without the pandemic symptoms**
**Hearing level**	0.068**	0.106**	0.142**	0.277**	−0.202**	−0.102**	−0.026	−0.059*

IES-R, Impact of Events Scale-Revised; *p < 0.05, ** *p* < 0.01.

Table 4 indicated that there was a significant effect between the hearing level and three preventive health measures except washing hands immediately after coughing, or sneezing, rubbing the nose (*R*^2^ =0.004 to 0.041, *p* < 0.05), which indicated that hearing disability was a higher risk factor for lower compliance with those preventative measures.

**Table 4:** The correlation between hearing level and the scores of four preventive health measures by the linear regression model.

	***R* ^2^**	**Unstandardized coefficients**		**Standardized coefficients beta**	***t***	***P*-value**
		**β**	**Std. Error**			
Avoiding sharing of utensils during mealtime	0.0409	−0.170	0.021	−0.202	−8.224	< 0.001
Washing hands with soap and water	0.0104	−0.0485	0.0119	−0.102	−4.088	< 0.001
Washing hands immediately after coughing, or sneezing, rubbing the nose	0.001	−0.016	0.015	−0.026	−1.033	0.302
Wearing masks with or without the pandemic symptoms	0.004	−0.024	0.010	−0.059	−2.370	0.018

### Mediating effect of mental health and psychological impacts between hearing level and the behavioral compliance with preventive health measures

**Figure 4:**
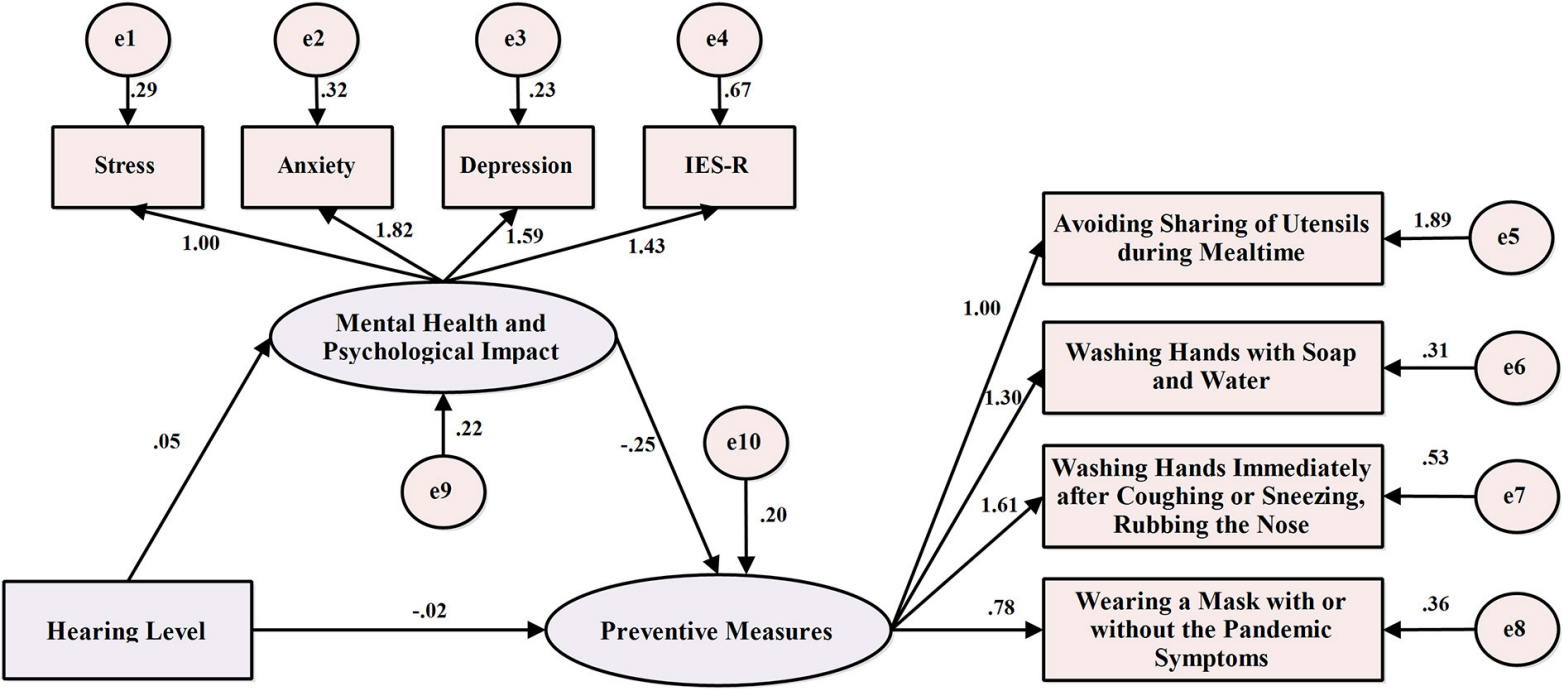
Structural equation model of hearing level, mental health status and psychological impacts, and the prevention scores of four preventive health measures.

Figure 4 shows a structural equation model with the hearing level as the independent variable, the preventive scores of four preventive health measures as the dependent variable, and mental health and psychological impacts as the intermediate variables. It was found that the path coefficients of the model were statistically significant (*p* < 0.05) and the fitting indexes were good, indicating that the model had been well-constructed (Table 5). Meanwhile, we used the bootstrap method to detect the mediating effect of mental health status and psychological impacts. Bootstrap repeat sampling was set to 2,000 and with 95% CI. The results showed that the direct effect was −0.073 (95% CI: −0.138 to −0.008), and the indirect effect was −0.044 (95% CI: −0.068 to −0.027) (Table 5). The study revealed that the direct and indirect effects of the hearing level on the prevention scores of four preventive health measures are statistically significant and 95% of the CIs did not include zero. Therefore, mental health and psychological impacts were shown to have a mediating effect on compliance with preventive health measures, and the mediating effect accounted for 37.60% of the total effect.

**Table 5:** Structural equation model fit index.

**Fit index**	**CMIN/DF**	**RMSEA**	**GFI**	**AGFI**	**TLI**	**IFI**	**CFI**	**PGFI**	**NFI**
Test result	7.902	0.066	0.974	0.953	0.933	0.954	0.953	0.541	0.947
Fit standard	1 < χ^2^/*df* < 3	< 0.08	>0.90	>0.90	>0.90	>0.90	>0.90	>0.05	>0.90

CMIN/DF, chi-square minimum degrees of freedom; RMSEA, root mean square error of approximation; GFI, goodness of fit index; AGFI, adjusted goodness of fit index; TLI, Tucker-Lewis index; IFI, incremental fit index; CFI, comparative fit index; PGFI, parsimonious goodness of fit index; NFI, normed fit index.

## Discussion

This study compares behavioral compliance with four preventive health measures between SHD and SNH during the COVID-19 pandemic in China. When the prevention scores of these two groups of students were examined in association with different demographic variables, mental health status and psychological impacts, the SHD consistently displayed a significantly lower degree of compliance with all four preventive health measures than SNH (Figures 1–3). Additionally, the direct and indirect effects of the hearing level on compliance with four preventive health measures are significant, and mental health and psychological impacts were shown to have a mediating effect on compliance with preventive health measures (Figure 4).

This finding is likely due to the difference in the demographic variables, mental health status and psychological impacts between these two groups of students. In contrast to SHD, a larger percentage of SNH has a better education background, satisfying with their current communication mode, doing exercise during the pandemic, and with a bedtime between 9 and 11 p.m. Most SNH is from a family with a steady income and their parents have a better educational background (Table 1). Furthermore, they also suffered less degree of pandemic-induced mental health issues and psychological impacts than SHD (Table 2).

A previous study has shown that optimization of infection management in health care with behavioral change can reduce the risk of infection during COVID-19 (28). The interventions designed to change behaviors are more effective if they target socialization factors. For this reason, individual practice and compliance with basic preventive health measures are essential during the pandemic.

Chinese people are accustomed to communal eating using chopsticks and spoons as the essential eating utensils. The eighth edition of China's diagnosis and treatment protocol for COVID-19 patients has explicitly recommended that COVID-19 patients should eat separate meals after discharge because the pandemic virus can be transmitted through shared cutlery (29, 30).

We found that the percentage of both groups of students with ≤ 2 high-risk prevention scores is far larger in the preventive health measure of avoiding sharing of utensils during mealtime than the other three preventive health measures in all demographic variables examined (Table 1). This finding suggests the deep influence of the traditional communal eating habit among all participants. The fact that the degree of compliance in this preventive health measure is lower in a large percentage of SHD may be due to a lack of understanding of the benefits of separate eating resulting from their limited communication strategies or stronger communal eating habit on the campus or at home.

The preventive health measure of hand hygiene is an acquired habit that is greatly associated with self-discipline and habituation (30). The practice of hand hygiene such as hand washing and alcohol-based hand rub can reduce the spread of respiratory infections and nosocomial infection rate (9). Previous studies have shown that young females are more inclined to avoid the risk of infection than males (31). In agreement with these studies, we have found that the degree of compliance with hand hygiene and wearing a mask for preventive health measures is consistently higher in a larger percentage of participants with >2 low-risk prevention scores for the female than the male in both groups of students (Figures 3B–D).

We also found that the degree of compliance with preventive health measures of mask-wearing is significantly lower for the female SHD than for female SNH. Since most female SHD (78.1%) use sign language and lip-reading for communication, the disadvantage of effective social communication created by mask-wearing may significantly reduce their willingness to comply with mask-wearing measures during the pandemic. This finding is corroborated by a recent study that shows hearing-impaired individuals who primarily use facial cues such as facial and lip expressions for social communication are less inclined to wear a mask during the pandemic (32).

The practice of proper mask-wearing is an effective non-pharmaceutical intervention in curtailing COVID-19 virus transmission (33). However, mask-wearing reduces a speaker's voice by 3–4 dB (surgical mask) or 12 dB (N95 mask) in the frequency range of 2,000–7,000 Hz (34). All these studies are supported by our finding that the degree of compliance with mask-wearing is significantly lower for the SHD than for the SNH because 373 (76.9%) SHD have profound hearing loss and 380 (78.4%) SHD rely on gesture and or lip-language or word for social communication. A recent study has shown that such a disadvantage in speech perception and communication induced by mask-wearing can be remedied by wearing a transparent mask (33).

On the other hand, one study has shown that parental education, socioeconomic conditions, and family structure play an important role in influencing adolescent health behavior (35). We have found that the parents' education is below senior high school and the parents' profession are jobless, self-employed, or farmers, their degree of compliance with three preventive health measures is significantly reduced for the SHD than for the SNH (Figures 1A,B,D).

A previous study indicated that male sex, rural residents, respondents with a low level of education, those engaged in agricultural, laboring, and domestic work, and people with disabilities were more likely to have difficulty practicing COVID-19 protective behaviors (36). It has been also reported that the unemployment rate may be high for the worker or employed staff when the parents with low education levels during the COVID-19 pandemic (37). Also, low-income families are prone to tension such that their children may be more susceptible to suffering from anxiety and depression status (37, 38). In agreement with these studies, we found that the SHD and the SNH, whose father's education level was below junior high school, mother's profession was a worker or employed, and with anxiety and depression, have a significantly lower degree of compliance with hand hygiene (Figure 3C).

Previous studies have shown that a lack of social interaction, communication, and access to public information resources produces negative impacts on deafness' mental health and psychological status (20, 39). In this study, we found that SHD consistently shows a significantly lower degree of compliance with the preventive measures of avoiding sharing utensils during mealtime and washing hands with soap and water than SNH regardless of their mental status and psychological impacts (Figures 2A,B). Meanwhile, hearing disability and anxiety status are risk factors for these health behaviors (Figures 3A,B). Conceivably, the SHD who inevitably have limited access to update pandemic information and those who suffer from anxiety are the key educational targets for the above two health behaviors.

It is worth mentioning that students aged below 18 years old performed much better compliance with wearing masks than those 18 years old and above. The majority of students aged 18 and older are already in college and may not be on the daily healthy behavior education as junior and senior students during the pandemic, these students should be the target group to focus on the wearing masks during the COVID-19 pandemic (Figure 3D).

It has been reported that outdoor exercise and limiting screen time can promote better mental and general health during the COVID-19 pandemic (40). In agreement with this finding, we found that students with a bedtime after 11 p.m. have poor hand hygiene behaviors, and physical activity is important in promoting all four preventive health habits (Figures 3A–D).

There was a correlation between hearing level, mental health status and psychological impacts, and the scores of the preventive health measures, which met the basic criteria of intermediary effect (Table 3). The association between hearing level and compliance with the three protective measures further supports our contention that hearing disability is a higher risk factor for lower compliance with preventative measures, and with a dose-dependent effect (Table 4).

Intermediary effect analysis showed that a higher degree of hearing disability and psychological distress can predict worse protective behavior compliance, supporting that hearing level, mental health status and psychological impacts are predictors of the preventive health measures. Meanwhile, mental health status and psychological impacts played an intermediary role between hearing level and compliance with preventive health measures. These results indicated that hearing level not only directly affected compliance with preventive health measures but also indirectly regulated compliance with preventive health measures through mental health status and psychological impacts (Figure 4).

The previous study of the general population at the initial stage of COVID-19 reported lower levels of psychological impacts, depression, anxiety, and stress were associated with higher compliance with precautionary measures (26), which is consistent with our findings on SHD. Our result also found that the higher the degree of hearing disability was, the worse the compliance with protective behavior would be. Furthermore, mental health status and psychological impacts indirectly regulate compliance with preventive health measures, clinical caregivers can indirectly improve the compliance with preventive health measures of people with hearing disability through the enhancement of psychological intervention during COVID-19.

Because of the disproportionate distribution of available COVID-19 vaccines, many parts of the world are still in great short supply of vaccines for effective treatments for the pandemic. It has been indicated that health literacy is an underestimated problem and mass practice of preventive health measures is crucial for the prevention of the spread of the pandemic (41). There is limited evidence on compliance with preventive measures for people with disabilities in the existing literature and our study provides a population perspective on behavioral strategies for hearing disability. As evident by the consistent lower degree of compliance with preventive health measures for SHD than for SNH, our study strongly suggests the importance of the development of health guidance and dissemination of updated pandemic information, essential communication services, extra assistance, and support to persons with a disability such as hearing loss who are more susceptible to a public health emergency and psychological distress.

This study has some limitations that require consideration. Firstly, our study was an online survey utilizing a snowball sampling method voluntarily. Therefore, many SHD did not participate in the questionnaire resulting in a large number of SNH participants and a small number of SHD participants. Secondly, most of the students were profound hearing disability in our study, and future studies will explore preventive health behavior in the general population with mild to moderate hearing loss. Lastly, our respondents are mainly students with or without hearing disability, the observation may not apply to the population in all social strata. Future research is necessary to survey participants from multiple geographic regions across all social strata.

## Conclusions

In conclusion, the SHD consistently shows a significantly lower degree of compliance in all four preventive health measures than SNH because of their negative emotional response, and inconvenient access to public information on the COVID-19 pandemic due to their physical disability. Hearing level and mental health status and psychological impacts are predictors of compliance with preventive health measures. Mental health status and psychological impacts have a partial mediating effect between hearing level and compliance with preventive health measures. At the same time, psychological support should also be provided to indirectly improve compliance with health behavior for people with hearing disability. To sum up, to reduce the risk of contraction, update pandemic information, essential communication services, extra assistance, and support should be provided to persons with a physical disability who are more susceptible to a public health emergency.

## Data availability statement

The raw data supporting the conclusions of this article will be made available by the authors, without undue reservation.

## Ethics statement

The studies involving human participants were reviewed and approved by Ethical approval was obtained from the Institutional Review Board of Binzhou Medical University (BMU-IRB-2020-54). Informed consent was obtained from each participant before taking the online survey. Written informed consent to participate in this study was provided by the participants' legal guardian/next of kin.

## Author contributions

YY, CQ, and PH-SJ designed the study concept, analytical methods, and presentation of the results and were responsible for data visualization. YY, YL, and YX obtained and managed data, implemented methods, and wrote the first draft of the paper. YY was responsible for project administration. YY and YL prepared original data. YY and PH-SJ supervised the study.YY, YL, YX, CQ, and PH-SJ reviewed and edited the manuscript. All authors helped with the interpretation of the results and approved the final manuscript.

## Funding

This work was supported by the Humanities and Social Sciences Youth Foundation, Ministry of Education of the People's Republic of China (18YJC740128), Natural Science Foundation of Shandong Province China (ZR2021MC052), Postgraduate Education Quality Improvement Plan in Shandong Province China (SDYAL19164), National College Students Innovation and Entrepreneurship Training Program (S202010440021), and Shandong Provincial Innovation and Entrepreneurship Training Program for College Students China (S202010440058).

## Conflict of interest

The authors declare that the research was conducted in the absence of any commercial or financial relationships that could be construed as a potential conflict of interest.

## Publisher's note

All claims expressed in this article are solely those of the authors and do not necessarily represent those of their affiliated organizations, or those of the publisher, the editors and the reviewers. Any product that may be evaluated in this article, or claim that may be made by its manufacturer, is not guaranteed or endorsed by the publisher.

## Abbreviations

SHD: students with hearing disability
SNH: students with normal hearing
DASS-21: Depression Anxiety and Stress 21
IES-R: Impact of Events Scale-Revised.
